# Iron bioavailibity from a tropical leafy vegetable in anaemic mice

**DOI:** 10.1186/1743-7075-8-9

**Published:** 2011-02-03

**Authors:** Fiona Hamlin, Gladys O Latunde-Dada

**Affiliations:** 1 King's College London, Diabetes and Nutritional Sciences Division, School of Medicine, Franklin-Wilkins Building, London, SE1 9NH, UK

## Abstract

*Telfairia occidentalis *is a vegetable food crop that is indigenous to West Africa. The leaves and seeds are the edible parts of the plant and are used in everyday meals by incorporation into soups and stews. Previous studies have attributed improved haematological indices to the vegetable and have advocated the use of *T. occidentalis *in the treatment of anemia. This study investigates the ameliorative effects of *T. occidentalis *when compared to FeSO_4 _as a reference salt in anaemic mice. It also compares the bioavailability of test iron and hepatic hepcidin expression for the estimation of iron absorption in the mice. Non-haem iron was determined in the liver of mice after the experimental feeding treatments. Hepcidin mRNA expression was carried out by quantitative RT-PCR. Administration of *T. occidentalis *leaves led to a modest increase in haemoglobin (Hb) levels in anaemic mice that were comparable to the Hb repletion in anaemic mice given FeSO_4. _Hepatic iron increase in the mice given either *T. occidentalis *or FeSO_4 _led to a corresponding enhancement of hepcidin mRNA expression. Induced hepcidin mRNA expression was enhanced by the addition of ascorbic acid to the test dose of iron. Hepatic hepcidin mRNA expression was found to be responsive to increase in the relative bioavailability of iron from test diets.

## Introduction

Iron deficiency anaemia is a nutritional disorder afflicting large population groups in the world. It is prevalent amongst vulnerable infants, adolescent girls and pregnant women particularly in populations subsisting largely on plant food sources. Remarkably, however, some leafy vegetables have been identified and used locally in treating anaemia. These include *Telfairia occidentalis *a member of the family Cucurbitaceae commonly known as the fluted gourd, fluted pumpkin or ugwu which is widely cultivated in countries such as Nigeria in West Africa.

Some investigators have proposed the use of *T. occidentalis *in the treatment of anaemia [1]. Studies have reported a number of haemoglobin (Hb) regeneration effects of the vegetable [2]. Osuntoki et al [3] reported the stabilization of human erythrocyte membranes in subjects given an extract of *T. occidentalis. *Moreover, *T. occidentalis *leaf protein incorporated in infant weaning foods had significant effects on iron status indices [4]. Olaniyan *et al *[1] served thirty anaemic pregnant women from rural communities with an oral mixture of *T. occidentalis *leaves, milk and raw egg three times a day for seven days and observed a significant rise in mean packed cell volume (PCV). Similarly, Oyedeji *et al *[5] served the *T. occidentalis *extract sweetened with milk to two severely anaemic paediatric patients and reported an increase in PCV. However, the reliability of these human studies is questionable, arising from the limited number of subjects, lack of statistical tests, poor study designs and limited use of haematological markers of iron status. Animal studies have been a useful source of evidence for understanding the effects of *T. occidentalis*. For example, Alada *et al *[6] studied haematological parameters and histological changes in the liver, intestine and testes of rats and observed hypertrophy of the intestinal propria and reduction in globlet cells. Haematological biomarkers are useful for investigating the nutritional status of mice and by comparing to normal values they can be used to determine the effect of the diet. Moreover, FeSO_4 _as in haemoglobin repletion assays was used as a reference salt of high bioavailability. It is against this background that the current study was conducted to determine the effect of *T. occidentalis *leaf extract on Hb regeneration response in iron-depleted mice. This study investigated further the molecular mechanisms of iron bioavailability measurement using this vegetable as an example.

## Materials and methods

### Reagents and Chemicals

Chemicals and biochemical reagents were of Analar grade and either from BDH-Merck Ltd., (Poole, Dorset, U.K.) or Sigma Chemical Company Ltd., (Poole, Dorset, U.K.).

### Plant Materials

Leaves of *Telfairia occidentalis *(100 g) were homogenized in a blender with de-ionised water on an equal volume per gram weight basis. Subsequently, the homogenate was filtered through layers of muslin cloth to obtain an extract. Samples of fresh leaves and their aqueous extracts were analysed using Association of Official Analytical Chemists [7] protocol for Fe analysis.

### Iron determination in the vegetable

*T. occidentalis *leaves and 10 ml of their extracts were weighed in crucibles with lids. The crucibles were labelled with porcelain marker and dried in an oven at 70°C overnight and cooled in a desiccator. The vegetables were charred over a bunsen burner at a low heat before placing them in a muffle furnace at 525°C for three hours during which all the organic matter was burnt leaving remnants of clean white ash. Samples were oven-dried for 48 hours, cooled in a desiccator and reweighed. Iron concentration in the samples was analysed using Inductively Coupled Plasma Optical Emission Spectrometry (ICP-OES), and values in ppm were extrapolated from the standard curve.

### Experimental Design and Animals

CD1 mice in groups of 4-8 were used for all experiments. Dietary iron deficiency was induced by feeding 3-weeks old CD1 male mice with a low-iron diet (Formula TD 80396, Harlan-Teklad; Madison, WI, U.S.A.; elemental iron concentration: 3 mg/kg) for 3 weeks. All mice were provided with food and water *ad libitum *throughout the experimental period.

Haematinic response of *T. occidentalis *was investigated by comparing with that induced by ferrous sulphate. Mice in groups of 4-8 were randomly allocated to the control group and two other experimental groups and administered the extract for 10 days. *T. occidentalis *extract and ferrous sulphate solution were formulated to contain 1.78 mg/L of iron. Mice were given fresh *T. occidentalis *extract or FeSO_4 _solution ad *libitum *daily and the record of daily consumption was taken. No precipitation was observed in the drinkers and total iron intake was equal among the experimental groups. Mice were maintained on low-iron diet during the duration of the experiment. After the experimental period, blood samples were collected and the mice were sacrificed for tissue collection. Liver samples were removed and snap-frozen in liquid nitrogen for subsequent analysis. A second set of experiment studied the effect of iron and ascorbic acid on liver iron levels and hepcidin mRNA expression in anaemic mice. Mice were grouped into control (normal mice), iron deficient mice, iron deficient mice administered iron (10 mg/L), and iron deficient mice administered iron (10 mg/L) + ascorbate (10 mg/L). Blood and liver samples were collected for determining haemoglobin (Hb), and liver tissues for analysis of iron and hepcidin mRNA expression studies.

All procedures were conducted in accordance with methods approved by the U.K. Animals (Scientific Procedures) Act, 1986.

### Haematologic and iron assays

Haemoglobin concentrations were calculated from the change in optical density at 540 nm, following the addition of 5 μl of whole-blood to Drabkin's reagent [8] and centrifugation (Heraeus Biofuge Pico, U.K.) at 13000 rpm for 5 minutes.

### Serum Iron Assay

Serum iron concentration of mice was determined using the QuantiChrom iron assay kit (DIFE-250, Bioassay Systems, USA) for quantitative colorimetric iron determination at 590 nm. The protocol was as described by the manufacturer.

### Ferritin Assay

The Spectro Ferritin MT Enzyme Linked Immunosorbent assay (ELISA) Kit was used for the ferritin assay. Serum ferritin concentrations were extrapolated from a standard curve as described in the manufacturer's protocol.

### Tissue Non-haem iron determination

Tissue non-haem iron levels were measured as previously described by Simpson and Peters [9]. Tissue samples were weighed and homogenized (1:5 w/v) in 0.15 M NaCl in 10 mM NaOH-Hepes buffer (pH 7.0) using a 1 ml glass dounce homogenizer (Wheaton Scientific, Millville, N.J., U.S.A.). An aliquot of the homogenate was then analysed for non-haem iron using a modification (Simpson & Peters, 1990 [9]) of the original method of Foy *et al *[10]. The iron values are expressed as either content (μmol Fe/organ) or concentration (nmol Fe/mg wet weight).

### Western blotting

The duodenal mucosa samples were scraped with a glass slide and homogenised (in a buffer containing 50 mM mannitol, 2 mM Hepes, 0·5 mM PMSF and pH 7·2) with an Ultra Turrax (IKA, Staufen, Germany) homogeniser (3 × 30 s pulses at full speed). The homogenate was centrifuged at 1500 ***g ***for 5 min and the supernatant was centrifuged for 1 h at 15 000 ***g ***to obtain the crude membrane fraction. Protein concentration was determined using Bio-Rad reagents (Bio-Rad Laboratories, U.S.A.). Fifty (50) μg of membrane extracts were loaded on a 12% gel in a Bio-Rad SDS-PAGE kit. The proteins separated were then transferred to Hybond ECL-nitrocellulose membrane (Amersham Biosciences, Bucks, UK) using a Bio-Rad semidry transfer apparatus (Bio-Rad Trans-Blot^R ^SD Semi-Dry Transfer Cell). Membrane was blocked with 5% milk for 1 h and probed with DMT1-IRE polyclonal, (Novus Biologicals) or β-actin (Sigma) antibodies diluted in 0.01% milk in TBS. Cross-reactivity was observed with peroxidase-linked anti-IgG by using SuperSignal West Pico (Thermo Scientific, U.S.A.).

### Real Time-PCR

Total RNA was isolated using the Trizol reagent (Invitrogen, Life Technologies, Paisley, U.K.) according to manufacturer's instructions. Quantitative RT-PCR was carried out using two-step ABI Prism 7000HT Sequence Detection System. First strand synthesis was performed using the ABI cDNA Synthesis Kit using 2 μg total RNA template according to the manufacturer's protocol. RNA quality was verified by running aliquot samples in formaldehyde gel that revealed distinct 18S and 28S ribosomal bands. In the second step, transcripts of the various genes were amplified with the specific primer sequences (Table 1) using the ABI SYBR Green supermix protocol. The efficacy of the amplification was confirmed by a melting curve analysis and gel electrophoresis to confirm the presence of a single product.

**Table 1:** Primer sequences used for quantitative real-time polymerase chain reaction analysis.

Primer	Sequence
mHepcidin 1-Forward	AGAGCTGCAGCCTTTGCAC
mHepcidin 1-Reverse	GAGGTCAGGATGTGGCTCTA
m18S-Forward	GAATTCCCAGTAAGTGGCGGG
m18S-Reverse	GGGCAGGGACTTAATCAACG

Quantitative measurement of each gene was derived from a standard curve constructed from known concentrations of PCR product. The results were calculated by the ∆Ct method that expresses the difference in threshold for the target gene relative to that of 18S ribosome. Sequences of gene specific primers were designed across intron-exon boundaries using the ABI design software. Forward and reverse primers sequences respectively are shown in Table 1.

### Statistical analysis

The values are presented as means ± standard error of the mean (SEM). One way ANOVA (analysis of variance) was used to determine whether treatment groups differed significantly with a post-hoc Tukey's test to establish specific differences in group means. Differences were considered significant when P < 0.05. Statistical evaluations were calculated with SPSS statistics 17.0.

## Results

### Haematological Indices in mice after dietary treatment

There was a significant increase (P < 0.05) in haemoglobin levels found in the mice administered ferrous sulphate when compared to the control group fed on iron-deficient diet (Table 2). This represents a 39.8% increase in haemoglobin levels when compared to the control mice. Haemoglobin levels in mice given *T. occidentalis *treatment was 23.0% higher than the control group. This difference, unlike that of FeSO_4, _was not statistically significant (P < 0.05). Mice maintained on control purified (normal) diet containing 48 ppm iron had Hb value of 17.50 ± 0.55. However, the average Hb maintenance efficiency of mice that were fed on the iron deficient (3 ppm iron) diet and given *T. occidentalis *extract was 58% relative to those that were given FeSO_4 _in this study. Ferritin levels in the mice administered *T. occidentalis *or FeSO_4_were not significantly different from that in the control group as the mice were anaemic and still repleting their haemoglobin concentration during the period of the study (Table 2). Although serum iron increased slightly in mice administered *T. occidentalis *or FeSO_4_, the differences were not significant (Table 2).

**Table 2:** Haemoglobin and tissue iron levels of mice administered *Telfairia occidentalis *extract or FeSO_4_.

	Control	*Telfairia occidentalis*	**FeSO**_**4**_
**Hb (dL)**	8.3 ± 0.7	10.2 ± 0.5	11.6 ± 1.1*
**Ferritin (ng/ml)**	41.0 ± 6.1	41.4 ± 8.1	38.8 ± 3.2
**Serum iron (ng/dL**)	457.3 ± 34.0	530.5 ± 42.2	612.3 ± 42.2

Statistical significance is indicated relative to the control treatment group, ** P < 0.05. Data are shown as means ± SEM, n = 4 replicates, typical of two experiments.

### Non-haem iron levels in the liver of mice after feeding T. *occidentalis* and FeSO_4 _diets

**Figure 1:**
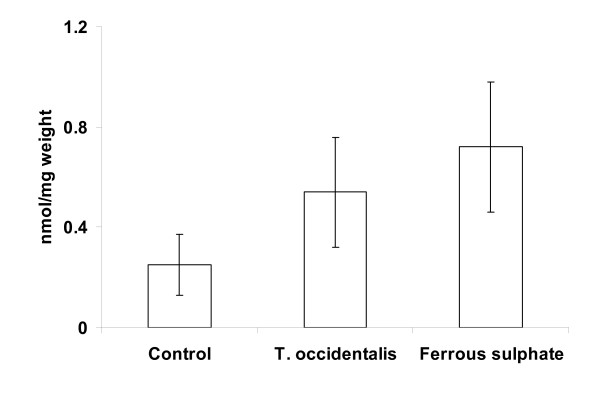
**Liver iron levels of anaemic mice administered *T. occidentalis *extract or FeSO_4 _solution for 10 days**. Data are shown as means ± SEM, n = 4 replicates, typical of two experiments.

The mean non-haem iron level in the liver was lowest in the control group when compared with mice administered *T. occidentalis *extracts or FeSO_4 _(Figure 1). The differences in non-haem iron levels between each treatment group were not statistically significant.

### Duodenal DMT1 protein levels of mice fed T. *occidentalis* and ferrous sulphate diets

**Figure 2:**
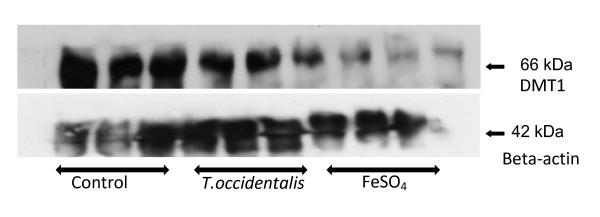
**Western blot analysis of DMT1 (divalent metal transporter 1) protein expression in duodenal extracts of mice administered *Telfairia occidentalis *or FeSO_4_**. 100 μg of protein was loaded for the triplicate samples. Beta-actin protein was probed on the blot as a loading control.

Western blot analysis of divalent metal transporter 1 (DMT1) protein expression in duodenal extracts of mice given *T. occidentalis *or FeSO_4_is shown in Figure 2. DMT1 protein expression was reduced in both treatment categories due to iron absorption in the duodenum of the mice.

### Effect of iron and ascorbic acid on liver iron levels and hepcidin expression

**Figure 3:**
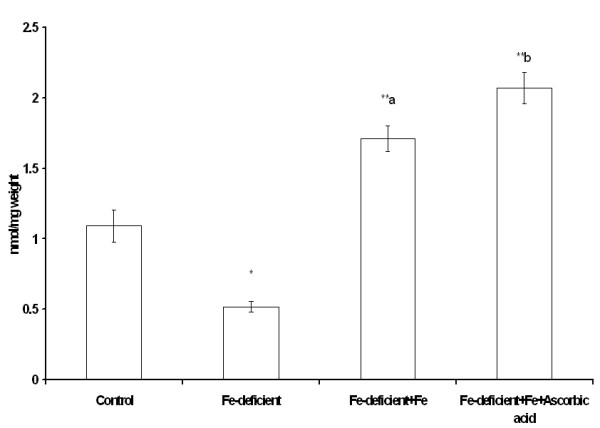
**Liver iron levels of anaemic mice administered FeSO_4 _and ascorbic acid solution for 10 days**. Statistical significance is indicated relative to the control treatment group, ** P < 0.05. Bars bearing different superscript letters are significantly different. Data are shown as means ± SEM, n = 4 replicates, typical of two experiments.

In order to ascertain the use of this approach in iron bioavailability studies, the effect of ascorbate, a potent enhancer of iron absorption, was studied. Haemoglobin levels after the experiment were 15.9, 14.4, 17.9 and 18.2 dL for mice in the control (normal diet), control (iron deficient diet), iron deficient + FeSO_4 _and iron deficient + FeSO_4 _+ ascorbic acid categories respectively. Iron and ascorbate administered jointly to anaemic mice significantly enhanced haemoglobin regeneration after 10 days. As expected, hepatic iron level was significantly reduced (P ≤ 0.05) after 3 week on the iron deficient diet. The administration of FeSO_4 _significantly increased liver iron in the mice. Moreover the addition of ascorbic acid enhanced (P ≤ 0.05) the increase in hepatic iron level (Figure 3).

### Hepatic hepcidin mRNA expression

**Figure 4:**
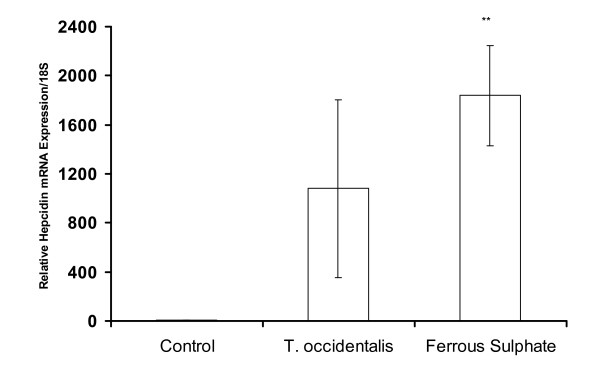
**Hepcidin mRNA expression in liver of anaemic mice administered *T. occidentalis *or FeSO_4 _solution for 10 days**. Statistical significance is indicated relative to the control treatment group, ** P < 0.05. Data are shown as means ± SEM, n = 4 replicates, typical of two experiments.

**Figure 5:**
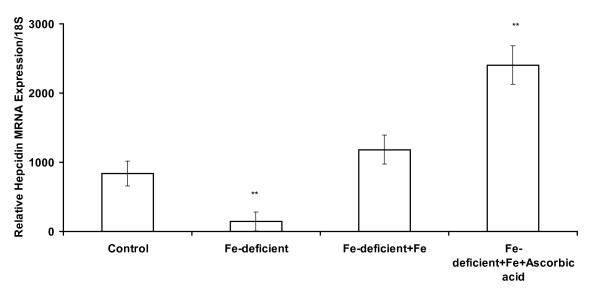
**Hepcidin mRNA expression in liver of mice administered FeSO_4 _and ascorbic acid solution for 10 days**. Statistical significance is indicated relative to the control treatment group, ** P < 0.05. Data are shown as means ± SEM, n = 4 replicates, typical of two experiments.

Hepcidin mRNA levels in the hepatic tissues of the mice in both sets of experiments mirrored liver iron levels (Figures 4 and 5). This observation demonstrates the responsiveness of hepcidin expression to iron modulation. It is reasonable to suggest a positive relationship between hepcidin expression in liver samples and the relative amount of bioavailable iron from the gastrointestinal tract of mice.

## Discussion

The iron content of the *T. occidentalis *fresh leaf samples used in this study was 0.6 mg/100 g, which was lower than the value (0.9 mg/100 g) reported by Fasuyi [11]. This leafy vegetable is commonly processed and added to stews and soups. Processing techniques such as blanching and homogenisation have been shown to significantly reduce the iron content of some vegetables [12]. The primary objective of the study was to compare the haemoglobin (Hb) regeneration response of anaemic mice to leaf extracts of *T. occidentalis *and FeSO_4 _using haemoglobin levels as a biomarker as compared to hepcidin mRNA expression.

Haemoglobin level is an acute response parameter and was shown to increase significantly, by 39.8% in the ferrous sulphate treatment group in this study when compared to control mice (Table 2). This finding is in agreement with the results of recent animal and human studies in which ferrous sulphate was effective as an oral iron supplement in the treatment of anaemia [13,14]. Ferrous sulphate is highly bioavailable when compared to other iron salts and it is used as a standard, after extrapolating to 100 percent, for measuring relative bioavailability of iron from foods. The use of iron salts as supplements and in food fortification is hindered by cost implications and sustainability in developing countries. Consequently, efforts have shifted towards the enhancement of iron bioavailability from food sources in alleviating the problem of iron deficiency anaemia. *Telfairia occidentalis *which is used in some traditional setting to treat anaemia exhibited a modest response in regenerating Hb level in the anaemic mice. In the current study, after 10 days of administration, *T. occidentalis *extract effected an iron bioavailability enhancement that was comparable to FeSO_4 _as judged by haemoglobin level. Emeka *et al*. [15] reported haemoglobin values of 12.8 g/dL when rats were fed *T. occidentalis *over a longer duration. Other studies have also shown that long-term intake of *T. occidentalis*-supplemented diet significantly increased haemoglobin concentration in rats [6]. Moreover, an air-dried *T. occidentalis *preparation significantly raised haemoglobin levels [16] in rabbits. This is presumably attributable to the supply of increased level of iron in a moderately bioavailable form. Thus *T. occidentalis *might be efficacious when administered over a long-term duration. In comparison, the current study had a shorter (10-day) duration. Moreover, its use as dried leaves will ensure significantly high intake of iron in soups and other traditional meals.

Studies investigating the use of vegetables in the treatment of anaemia measured Hb regeneration using haematological indices such as haemoglobin level, red blood cell count and packed cell volume [1,17]. These parameters respond to changes within a short duration of dietary treatment. Changes in serum iron and tissue iron, although not statistically significant, showed a similar trend to Hb levels when anaemic mice were given *T. occidentalis *or FeSO_4 _treatment (Figure 1). Changes in serum iron and iron parameters (such as ferritin that was measured in this study) are expected to be more significant with longer exposure of anaemic mice to iron administration.

Protein expression of the non-haem iron transporter, DMT1 (divalent metal transporter 1) responded to iron exposure in the duodenum of mice given *T. occidentalis *or ferrous sulphate (Figure 2). DMT1 expression is regulated via the transcriptional modulation of the iron response element and iron regulatory protein (IRE-IRP) mechanism [18]. Duodenal and hepatic iron levels tended to increase in both treatment groups. Administration of *T. occidentalis *was not as efficacious as FeSO_4 _judging by the parameters of Hb level, DMT1 down regulation and hepatic hepcidin elevation used in this study. This might be due to the presence of inhibitors such as polyphenols and fibre in the vegetable. Processing techniques that prevent loss of ascorbic acid in the vegetable could enhance iron bioavailabilty. Furthermore, there are differences in iron absorption regimes that limit extrapolation of results from mice studies on to human subjects [19].

Hepatic iron levels increased in mice after ten days of iron administration (Figure 3) in this study. Analysis of serum iron increase after meal intake has been advocated as an efficient method of comparing post-prandial iron absorption between groups in humans [20]. Recent advances in iron molecular research and the discovery of hepcidin have introduced templates that can be considered as indices of functional food bioavailability [21]. Plasma hepcidin, is an obvious non-invasive biomarker correlates strongly with serum ferritin that is analysed routinely in most laboratories. Differences in iron absorption were accounted for by variation in circulating plasma hepcidin [22,23]. However, it is expensive. In the current study, Hb repletion and hepatic hepcidin mRNA expression exhibited a trend that reflected responsiveness to bioavailability of the test doses in mice (Figures 4 and 5). It was observed in humans that differences in diets that vary in bioavailability are apparent in iron-deficient rather than in iron-replete subjects [24]. Moreover, iron absorption in the rat was shown to be dependent on serum iron levels rather than iron turnover or storage, as is the case in humans [25]. It is not known if the increase in hepcidin expression in anaemic mice is a quantitative function of serum iron increase after a test dose of iron. Furthermore, enhanced hepcidin expression will exert a negative feedback regulation on iron absorption which will culminate in the degradation of ferroportin over time. As with haemoglobin repletion assay, a pivotal issue is equimolar concentration of iron in test diets to ensure that relative bioavailability is comparable. This was ascertained in the current study as equal amounts of iron were ingested by mice in the different experimental groups. Consequently, the measurement of bioavailable iron will be influenced by the relative amounts of enhancers and inhibitors interacting in the lumen of the gastrointestinal tract.

Dietary inulin was shown to influence enterocyte expression of genes such as DMT1, Dcytb (duodenal cytochrome b) and ferroportin [26] which encode iron transport proteins. Dietary and luminal components exert effects on mucosa gene expression indirectly through their modulation of available iron [27]. Although different forms of iron can influence the expression of their respective transporters in the apical duodenum, systemic homeostatic mechanism might be rather complex and multifactorial [28]. Further research is needed in optimising the doses, duration and the kinetics of these variables in iron nutrogenomic and metabolomic studies.

Haemoglobin repletion was higher with FeSO_4 _than with *T. occidentalis *extracts in the current study. However, as problems arising from gastric and intestinal intolerance are manifest in large numbers of iron deficiency anaemia (IDA) patients administered with FeSO_4 _or other forms of redox-active iron, less reactive sources of the nutrient, such as from plants, are required as an alternative. Judging by our results, it is reasonable to suggest that *T. occidentalis *extract could be adopted in an alternative, longer-term, plant food-based supplementation strategy for IDA treatment.

## Competing interests

The authors declare that they have no competing interests.

## Authors' contributions

FH performed the dietary iron analysis, haematological assays and statistical analysis and GOLD carried out the gene expression studies. Both authors had input into the writing of the manuscript, read and approved the final manuscript.
